# Abnormal Brain Iron Homeostasis in Human and Animal Prion Disorders

**DOI:** 10.1371/journal.ppat.1000336

**Published:** 2009-03-13

**Authors:** Ajay Singh, Alfred Orina Isaac, Xiu Luo, Maradumane L. Mohan, Mark L. Cohen, Fusong Chen, Qingzhong Kong, Jason Bartz, Neena Singh

**Affiliations:** 1 Department of Pathology, Case Western Reserve University, Cleveland, Ohio, United States of America; 2 Department of Medical Microbiology and Immunology, Creighton University, Omaha, Nebraska, United States of America; University of Alberta, Canada

## Abstract

Neurotoxicity in all prion disorders is believed to result from the accumulation of PrP-scrapie (PrP^Sc^), a β-sheet rich isoform of a normal cell-surface glycoprotein, the prion protein (PrP^C^). Limited reports suggest imbalance of brain iron homeostasis as a significant associated cause of neurotoxicity in prion-infected cell and mouse models. However, systematic studies on the generality of this phenomenon and the underlying mechanism(s) leading to iron dyshomeostasis in diseased brains are lacking. In this report, we demonstrate that prion disease–affected human, hamster, and mouse brains show increased total and redox-active Fe (II) iron, and a paradoxical increase in major iron uptake proteins transferrin (Tf) and transferrin receptor (TfR) at the end stage of disease. Furthermore, examination of scrapie-inoculated hamster brains at different timepoints following infection shows increased levels of Tf with time, suggesting increasing iron deficiency with disease progression. Sporadic Creutzfeldt-Jakob disease (sCJD)–affected human brains show a similar increase in total iron and a direct correlation between PrP and Tf levels, implicating PrP^Sc^ as the underlying cause of iron deficiency. Increased binding of Tf to the cerebellar Purkinje cell neurons of sCJD brains further indicates upregulation of TfR and a phenotype of neuronal iron deficiency in diseased brains despite increased iron levels. The likely cause of this phenotype is sequestration of iron in brain ferritin that becomes detergent-insoluble in PrP^Sc^-infected cell lines and sCJD brain homogenates. These results suggest that sequestration of iron in PrP^Sc^–ferritin complexes induces a state of iron bio-insufficiency in prion disease–affected brains, resulting in increased uptake and a state of iron dyshomeostasis. An additional unexpected observation is the resistance of Tf to digestion by proteinase-K, providing a reliable marker for iron levels in postmortem human brains. These data implicate redox-iron in prion disease–associated neurotoxicity, a novel observation with significant implications for prion disease pathogenesis.

## Author Summary

Prion disorders are neurodegenerative conditions of humans and animals that are invariably fatal. The main agent responsible for neurotoxicity in all prion disorders is PrP-scrapie (PrP^Sc^), a β-sheet rich isoform of a normal cell-surface glycoprotein, the prion protein (PrP^C^). Deposits of PrP^Sc^ in the brain parenchyma are believed to induce neurotoxicity, though the underlying mechanisms are not entirely clear. Emerging evidence from prion-infected cell and mouse models implicates redox-iron in prion disease–associated neurotoxicity. However, a systematic evaluation of iron homeostasis in prion disease–affected brains and the underlying mechanism of iron dyshomeostasis are lacking. In this report, we demonstrate that prion disease–affected human, mouse, and hamster brains exhibit a state of iron deficiency in the presence of excess total brain iron, resulting in a state of iron imbalance. The underlying cause of this phenotype is likely sequestration of iron in cellular ferritin that becomes detergent-insoluble, possibly due to association with PrP^Sc^. This results in a state of iron bio-insufficiency, leading to increased iron uptake by the cells and worsening of the state of iron imbalance. Since iron is highly toxic if mismanaged, these results implicate iron imbalance as a significant contributing factor in prion disease–associated neurotoxicity.

## Introduction

Imbalance of brain iron homeostasis is considered an important contributing factor of neurotoxicity in several neurodegenerative disorders including Parkinson's disease, Alzheimer's disease, and Huntington's disease [1]–[4]. Recent evidence suggests a similar alteration of iron homeostasis in prion disorders, a group of neurodegenerative conditions affecting humans and animals [5]–[10]. Since prion disorders are believed to result from a change in the conformation of cellular prion protein (PrP^C^) from an α-helical to a β-sheet rich PrP-scrapie form (PrP^Sc^), disturbance of brain iron homeostasis is an unexpected outcome [11],[12]. Direct demonstration of increased total iron including Fe^2+^ and Fe^3+^ ions in the cerebral cortex, striatum, and brain stem of scrapie-infected mice, and an increase in markers of oxidative stress such as free malondialdehyde in diseased brains supports a role for redox-iron in prion disease pathogenesis, though the generality of this phenomenon and the underlying cause remain unidentified [5],[6],[10],[13].

Bearing in mind the pathophysiology of prion disorders, it is difficult to explain disruption of iron metabolism by the conversion of PrP^C^ to PrP^Sc^, the principal event in all prion disorders. Under normal conditions, cellular iron homeostasis is maintained by a set of iron regulatory proteins that respond to intracellular iron levels by regulating their expression. In iron deficient conditions, iron uptake proteins transferrin (Tf) and transferrin receptor (TfR) are up regulated, and the iron storage protein ferritin is down regulated. The net result is an increase in the bio-available labile iron pool. The opposite scenario takes effect when iron is in excess [14]. A tight regulation of cellular iron is important since iron is not only an essential component of enzymes and proteins required for optimal neuronal growth and function, but is also toxic due to its ability to exist in two oxidation states, ferric (Fe^3+^) and ferrous (Fe^2+^) [14]–[16]. Under conditions where ferritin is unable to detoxify iron, Fe^2+^ iron can participate in one-electron transfer reactions such as Fenton or Haber-Weiss' reactions, resulting in cell death. Thus, alteration of brain iron homeostasis in diseased brains is likely to induce significant neurotoxicity, an observation that has received little attention [16].

Although the origin of iron imbalance in diseased brains is unclear, *in vitro* studies conducted previously provide some clues to the underlying mechanism(s). It is likely that the affinity of PrP^C^ for iron and hemin and the influence of this interaction on its conformational change to PrP^Sc^ renders iron inaccessible from PrP^Sc^ aggregates [17],[18]. This would increase total cellular iron while decreasing the bio-available iron pool, inducing a state of cellular iron deficiency in the presence of excess iron. Alternately, since PrP is involved in cellular iron uptake and transport, a disruption of this function by aggregation of PrP to the PrP^Sc^ form may alter cellular iron homeostasis, creating a state of iron imbalance [19]. Though plausible, this phenomenon has not been evaluated in prion disease affected brains.

To fill this gap, we examined a large sample size of sporadic Creutzfeldt-Jakob disease (sCJD) affected human brains, scrapie infected mouse brains inoculated with the 139A strain [20], and hamster adapted Transmissible Mink Encephalopathy (TME) or 263K scrapie inoculated hamster brains to investigate the presence of iron imbalance during disease progression and at end-stage of disease [21]. In addition to measuring brain iron levels, particular attention was directed to Tf, the iron uptake protein that responds to minor changes in cellular iron levels, is unaltered by disease associated astrogliosis [2], [14]–[16], and resists harsh protease digestion (see below), making it possible to assess iron homeostasis in post-mortem human brains. Snap frozen mouse and hamster brains served as additional controls to rule out any effects of post-mortem interval in the human tissue. In addition, cell models that replicate prions *in vitro* were used to understand the underlying cause of altered iron homeostasis due to prion infection [22],[23]. We report that diseased human, hamster, and mouse brains show signs of iron imbalance, confirming the generality of this abnormality regardless of the prion strain or the affected species. The alteration of brain iron status appears early in the disease process, concomitant with the appearance of PrP^Sc^, and is not an outcome of end-stage disease. Furthermore, we demonstrate the sequestration of iron in detergent insoluble PrP^Sc^-ferritin complexes in PrP^Sc^ infected cell models and in sCJD brain homogenates, explaining the cause of iron imbalance in prion disease affected brains.

## Results

### Prion disease–infected hamster brains show evidence of brain iron imbalance

**Figure 1:**
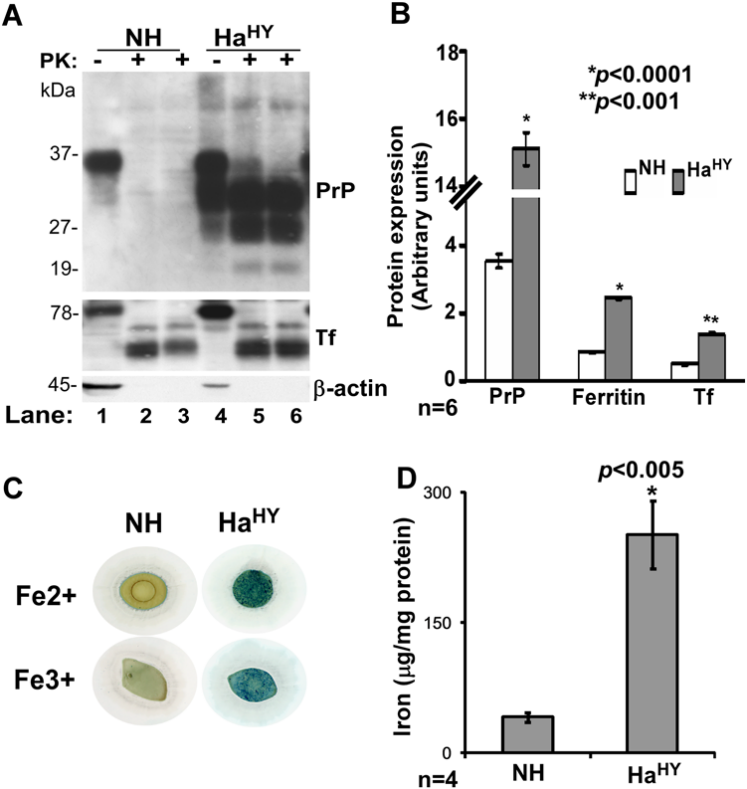
TME-infected hamster brains show alteration of brain iron homeostasis. (A) Normal (NH) and Ha^HY^-infected hamster brains harvested at the end stage of disease were homogenized, digested with 50 and 100 µg/ml of PK, and fractionated on SDS-PAGE, followed by immunoblotting. PrP from NH is degraded completely by this treatment (lanes 1–3), whereas diseased brains show typical PK-resistant PrP bands (lanes 4–6). Probing for Tf shows increased expression in the diseased sample (lanes 1 and 4). Surprisingly, Tf from both normal and diseased samples is partially cleaved by this treatment and resists further degradation (lanes 2, 3, 5, 6). (B) Quantitative analysis of six different undigested samples after normalization with β-actin shows a significant increase in PrP, Tf, and ferritin expression in the diseased sample compared to matched controls. Values are mean±SEM. *p<0.0001, **p<0.001 relative to NH. n = 6. (C) Equal protein from NH and Ha^HY^ brain homogenates was dot blotted on a PVDF and reacted for Fe(II) and Fe(III) iron. Ha^HY^ sample shows significantly more reactivity for Fe(II) and Fe(III) iron compared to the NH sample. (D) Estimation of total iron shows significantly more iron in the Ha^HY^ sample compared to matched NH samples. Values are mean±SEM. *p<0.005 relative to NH. n = 4.

To evaluate if prion infection induces imbalance in brain iron homeostasis, brain tissue from control (NH) and end-stage diseased hamsters inoculated with the hyper strain (HY) of hamster adapted TME (Ha^HY^) [17] was assessed for PrP^Sc^ levels, total iron, redox-active ferrous iron, and levels of major iron management proteins Tf and ferritin. Accordingly, NH and Ha^HY^ brain homogenates were digested with proteinase-K (PK) and analyzed by SDS-PAGE and immunoblotting. Probing with anti-PrP antibody 3F4 revealed the expected glycoforms of PrP^C^ comprising of unglycosylated and two glycosylated forms migrating between 27 and 37 kDa in the NH sample (Figure 1A, lane 1). Ha^HY^ showed significantly more reactivity and additional bands migrating between 19 and 37 kDa, typical of PrP^Sc^ (Figure 1A, lane 4). Treatment with 50 and 100 µg/ml of PK digested all PrP^C^ in NH (Figure 1A, lanes 2 and 3), whereas Ha^HY^ showed typical PK-resistant PrP^Sc^ forms migrating between 19 and 30 kDa (Figure 1A, lanes 5 and 6). Probing for Tf revealed relatively higher expression in Ha^HY^ compared to NH (Figure 1A, lanes 1 and 4), and surprisingly, partially cleaved PK-resistant Tf forms that migrated ∼10 kDa faster in both NH and Ha^HY^ samples (Figure 1A, lanes 2, 3, 5, 6). Similar resistance to PK was noted in commercially available human Tf and normal and sCJD affected human brain tissue (see below). Immunoblotting for ferritin showed a significant increase in Ha^HY^ infected samples as observed for Tf (data not shown). Quantitative estimation of PrP, Tf, and ferritin after normalization with β-actin showed an increase of 4.3, 2.8, and 2.9 fold respectively in Ha^HY^ compared to matched NH samples (Figure 1B). (β-actin is cleaved by PK (Figure 1A, lanes 2, 3, 5, 6). Estimation of ferrous (Fe (II)) and ferric (Fe (III)) iron showed significantly more reactivity in Ha^HY^ samples compared to matched NH samples as reported earlier for diseased human and mouse brains (Figure 1C) [16]. Total iron was increased by 4.9 fold in the Ha^HY^ sample relative to matched NH samples (Figure 1D).

### A similar evidence of iron imbalance is detected in scrapie-infected mouse brains

**Figure 2:**
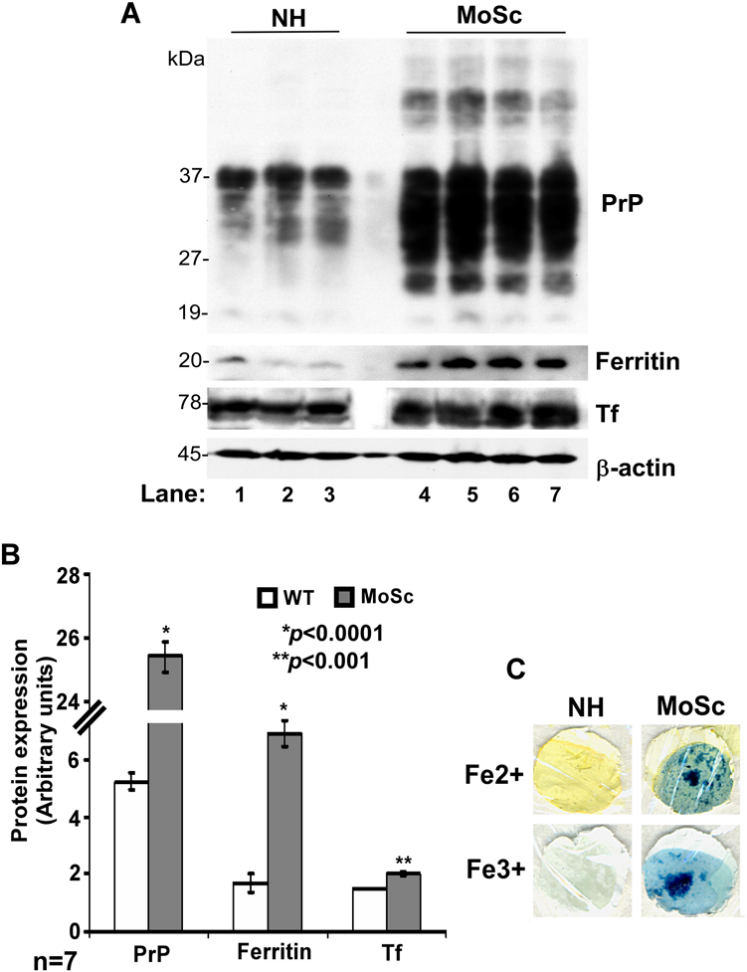
Scrapie-infected mouse brains show alteration of iron homeostasis. (A) Normal (NH) and scrapie-infected (MoSc) mouse brains collected at end stage of disease were homogenized and fractionated by SDS-PAGE, followed by immunoblotting. Probing with specific antibodies shows increased expression of PrP, ferritin, and Tf in MoSc samples compared to matched controls (lanes 1–7). (B) Quantitative analysis after normalization with β-actin shows a significant increase in PrP, ferritin, and Tf expression in diseased samples compared to matched controls. Values are mean±SEM. *p<0.0001, **p<0.001 relative to NH samples. n = 7. (C) Dot blot analysis as in Figure 1C shows a prominent reaction for Fe (II) and Fe (III) iron in MoSc homogenates compared to matched controls.

A similar analysis of normal (NH) and scrapie infected end stage mouse (MoSc) brain tissue showed increased accumulation of PrP and up regulation of ferritin and Tf in MoSc samples compared to matched controls (Figure 2A, lanes 1–3 and 4–7). Quantitative estimation of PrP, ferritin, and Tf showed an increase of 4.9, 3.9, and 1.4 fold respectively in MoSc compared to matched controls (Figure 2B). In addition, MoSc samples revealed increased reactivity for both Fe (II) and Fe (III) iron compared to matched controls (Figure 2C) as noted for hamster samples above and reported previously [18].

### Autopsy and biopsy samples of sCJD brains show similar evidence of iron imbalance

**Figure 3:**
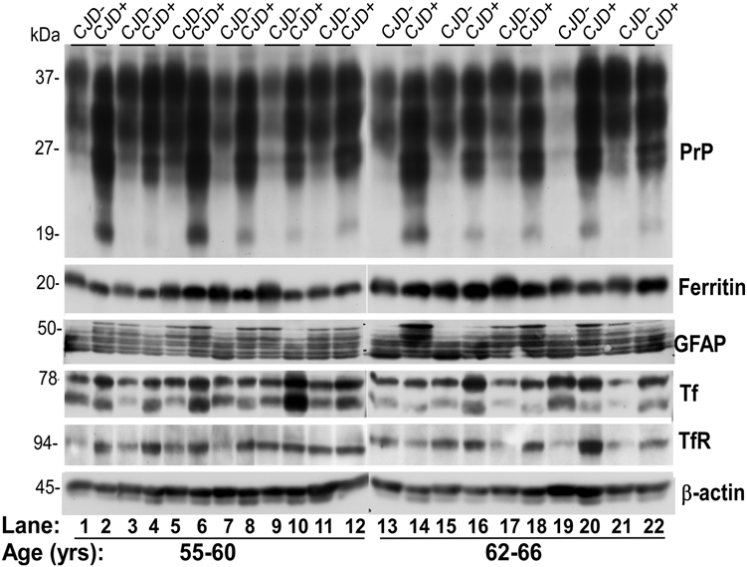
sCJD human brain homogenates show evidence of iron imbalance. Autopsy tissue from the frontal cortex of CJD+ and matched controls (CJD−) was homogenized and analyzed. Immunoblots of the first 11 cases are shown. Fractionation on SDS-PAGE followed by immunoblotting shows an increase in PrP, Tf, and TfR expression in CJD+ samples (even lanes) compared to matched controls (odd lanes). Ferritin and GFAP show an inconsistent difference between the two groups (lanes 1–22).

To evaluate if diseased human brains show a similar phenotype of iron imbalance, brain homogenates from 20 cases of sCJD (CJD+) and matched controls (CJD−) were fractionated by SDS-PAGE and immunoblotted as above. Probing for PrP showed the expected glycoforms migrating between 27 and 37 kDa in CJD− samples (Figure 3, odd lanes). CJD+ samples showed relatively more reactivity for PrP and an additional 19–20 kDa band typical of PrP^Sc^ (Figure 3, even lanes). Re-probing for ferritin and glial fibrillary acidic protein (GFAP), a marker for glial cells, showed an inconsistent difference between CJD− and CJD+ samples (Figure 3, odd and even lanes). However, Tf and TfR revealed a consistent increase in CJD+ samples compared to age-matched controls (Figure 3, odd and even lanes).

**Figure 4:**
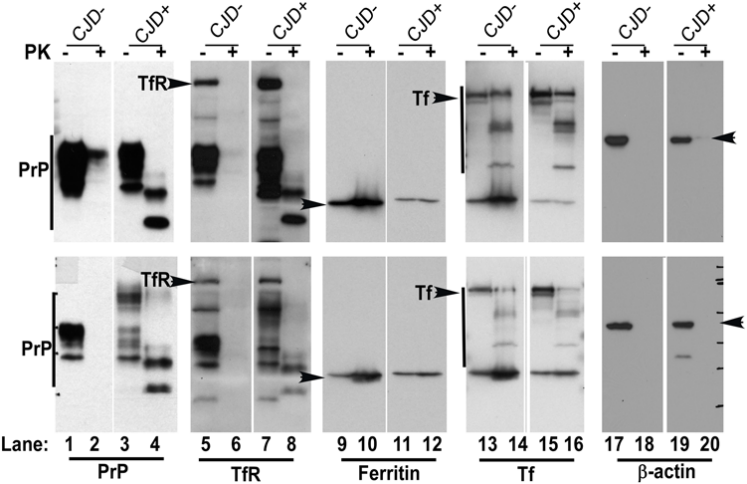
Biopsy samples of sCJD cases show similar evidence of brain iron imbalance. Representative PVDF membranes used previously to diagnose CJD− and CJD+ biopsy samples were re-probed sequentially for TfR, ferritin, Tf, and β-actin. (CJD− and CJD+ samples matched for age and disease duration are aligned for easier readout). Odd lanes show untreated controls, while even lanes show PK-treated samples. In CJD− samples, PrP, TfR, and β-actin are completely degraded by PK treatment (lanes 2, 6, and 18), whereas CJD+ samples show typical PK-resistant forms of PrP^Sc^ (lanes 4 and 8). Surprisingly, ferritin and Tf resist PK digestion in both CJD− and CJD+ samples (lanes 9–16). CJD+ samples show an increase in TfR and Tf levels relative to matched CJD− samples (lanes 5–8 and 13–16, respectively). Ferritin shows little difference between the two samples (lanes 9–12).

**Figure 5:**
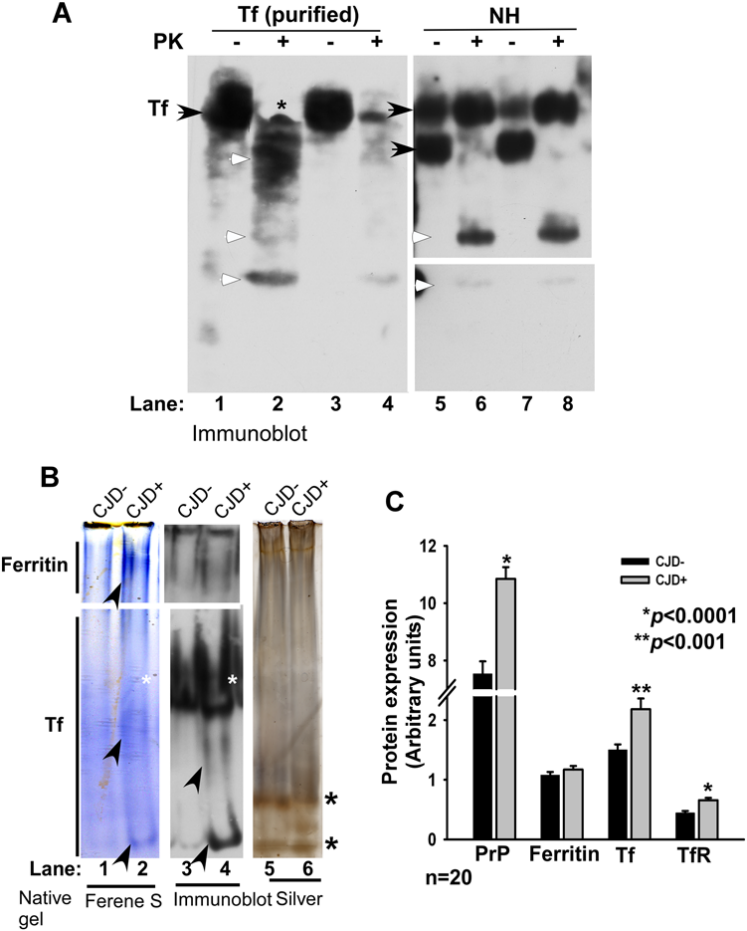
Purified and human brain Tf is PK-resistant. (A) Purified Tf (Sigma) and CJD− brain homogenate were digested with 50 and 100 µg/ml of PK and fractionated on SDS-PAGE, followed by immunoblotting. Purified and brain Tf are cleaved to faster migrating PK-resistant forms (lanes 1–8). (B) Samples from representative CJD− and CJD+ cases were fractionated on non-denaturing gel and stained with the iron binding dye ferene-S (lanes 1 and 2), or transblotted and probed for ferritin and Tf (lanes 3 and 4). CJD+ sample shows increased reaction for iron in ferritin and Tf (lanes 1–4). Lack of iron reactivity by one of the oligomeric Tf forms may be due to inaccessibility of iron to ferene-S (lanes 2 and 4, white asterisk). Silver staining of the same membrane confirms equal loading of protein in the two samples (lanes 5 and 6). (C) Quantification of autopsy samples analyzed by immunoblotting shows increased expression of PrP, Tf, and TfR in CJD+ samples relative to matched controls. Values are mean±SEM. *p<0.0001, **p<0.001 relative to CJD− samples. n = 40.

Since autopsy samples represent end-stage disease and carry the additional risk of artifacts due to post-mortem interval, a similar evaluation was carried out on human biopsy samples. Due to limited amount of biopsy tissue, PVDF membranes used for diagnostic purposes were utilized. Membranes from two representative CJD positive cases containing control (CJD−) and CJD positive (CJD+) samples either left untreated or subjected to PK treatment were reprobed for TfR, ferritin, Tf, and β-actin (Figure 4, odd and even lanes, respectively). As noted for autopsy samples, CJD+ biopsy samples demonstrated an increase in TfR and Tf levels, and an insignificant difference in ferritin levels compared to age-matched CJD− controls after normalization with β-actin (Figure 4, lanes 5–8, 13–16, 9–12). These results provide confidence that the differences between CJD− and CJD+ samples are not influenced by post-mortem interval. As observed for diseased hamsters, Tf showed resistance to PK digestion in CJD− and CJD+ samples (Figure 4, lanes 14 and 16), a novel finding that could prove useful in assessing the iron status of healthy and diseased post-mortem brains. This observation was therefore further validated by treating 10 and 5 ng of purified Tf isolated from human plasma with 50 µg/ml of PK for 30 minutes at 37°C in the presence of non-ionic detergents (Figure 5A, lanes 1–4). An aliquot (5 µl) of CJD− brain homogenate was processed in parallel (Figure 5A, lanes 5–8). Purified Tf migrated as a single band (Figure 5A, lanes 1 and 3, black arrow) and was cleaved to faster migrating forms when exposed to PK (Figure 5A, lanes 2 and 4, open arrowhead). Brain Tf migrates as a doublet, probably representing glycoforms of Tf (Figure 5A, lanes 5 and 7, black arrow), and is cleaved to faster migrating forms by PK treatment that co-migrate with similarly treated fragments of purified Tf (Figure 5A, lanes 6 and 8, open arrowhead). A similar resistance to PK has been reported for human brain ferritin [18].

To determine if the increase in Tf levels in CJD+ samples is accompanied by an increase in its iron content, CJD− and CJD+ samples were separated on a non-denaturing gel in duplicate. Proteins were either reacted with Ferene-S, a dye that forms a blue reaction product with iron (Figure 5B, lanes 1 and 2) [24], or transblotted to a PVDF membrane and probed for ferritin and Tf (Figure 5B, lanes 3 and 4), followed by silver staining of the membrane (Figure 5B, lanes 5 and 6). An increase in the level of ferritin and Tf expression and reactivity for iron was detected in the CJD+ sample (Figure 5B, lanes 1–4, black arrowheads). The slower migrating Tf reactive band probably represents a complex or glycosylated form of Tf that does not react with ferene-S for reasons that are unclear from our data (Figure 5B, lanes 2 and 4, white asterisk). Separation of purified ^59^Fe labeled Tf on a similar gel system reveals three labeled bands, though it is difficult to conclude which of the Tf bands from brain homogenates correspond to the isoforms of ^59^Fe-Tf [19]. Silver staining of the membrane shows two prominent unidentified bands in CJD− and CJD+ samples, and equal loading of protein in both samples (Figure 5B, lanes 5 and 6, black asterisk).

**Figure 6:**
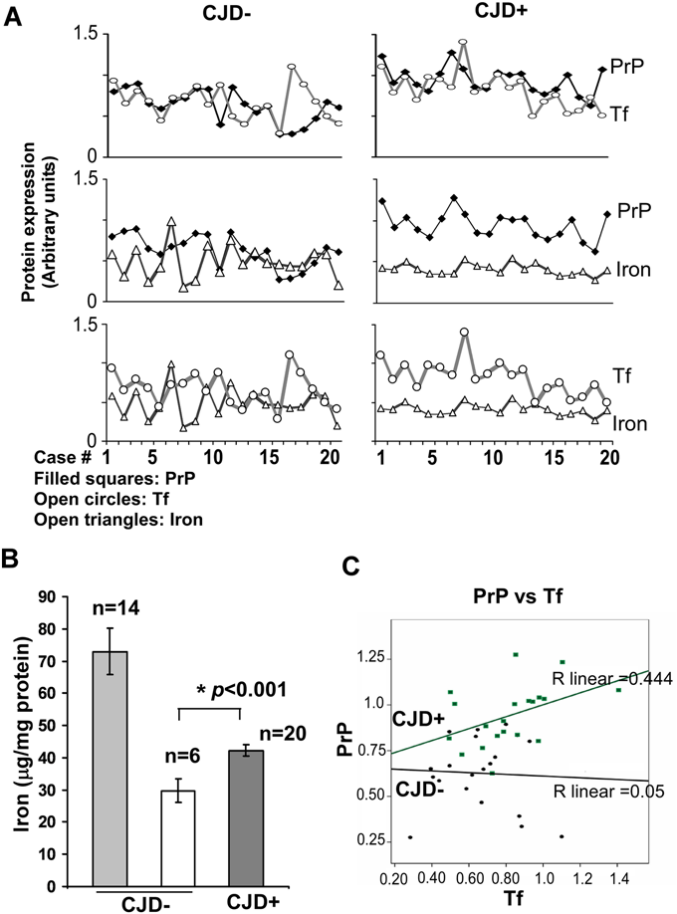
Correlation and linear reggression analysis of human autopsy samples. (A) Correlation between PrP and different proteins shows a strong positive correlation between PrP and Tf, PrP and iron, and Tf and iron in CJD+ cases. CJD− cases show no correlation between PrP and Tf and between iron and Tf, and modest correlation between PrP and iron. (B) Quantification of total iron shows two distinct groups of CJD− cases with high and low iron content, the higher values arising most likely from specific disease conditions (see Materials and Methods). In contrast, CJD+ cases show consistent iron levels in all 20 cases that are significantly higher than the apparently “disease-free” control group. Values are mean±SEM. *p<0.001. n = 20. (C) Linear regression analysis shows a positive correlation between PrP and Tf in CJD+ cases (R linear = 0.444 for CJD+, and 0.05 for CJD−). n = 20.

**Figure 7:**
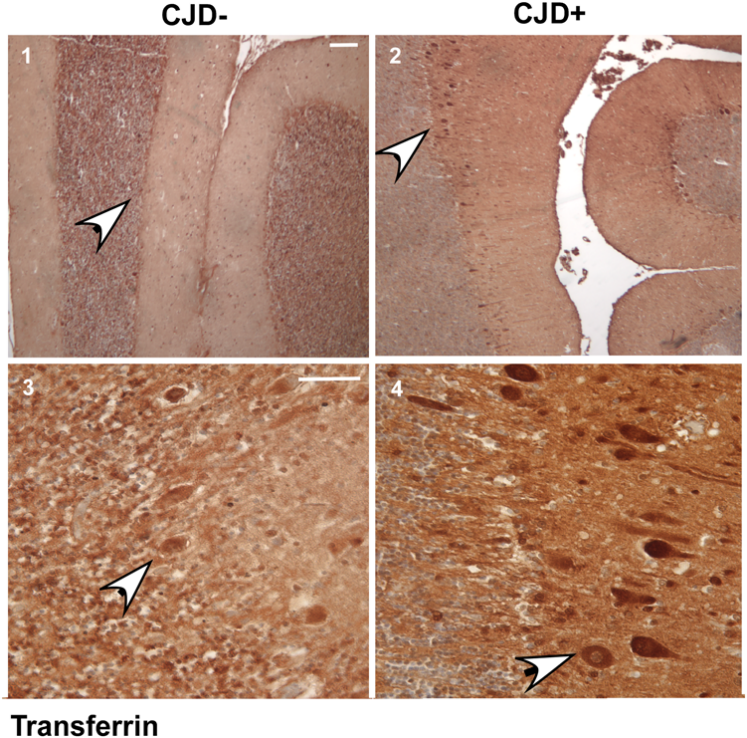
Immunoreaction of normal and sCJD brain sections for Tf. Brain sections from CJD+ and matched controls were immunostained with anti-Tf followed by DAB reaction. Purkinje cell neurons of CJD+ sections show strong reactivity for Tf. Low magnification images emphasize that the difference in Tf reactivity is not limited to a few neurons (upper panels). Bar: 10 µm. n = 3.

Quantitative analysis of 20 CJD+ and an equal number of age-matched CJD− autopsy samples showed a significant increase in PrP, Tf, and TfR levels in CJD+ samples by 1.5, 1.6, and 1.5 fold respectively. The difference in ferritin levels between CJD− and CJD+ samples is statistically insignificant (Figure 5C). Variability in the levels of different proteins within CJD− and CJD+ samples is shown in Figure 6A. The correlation between PrP and Tf was 0.05 in CJD− samples, and significantly higher at 0.444 in CJD+ samples. A similar assessment of PrP and iron revealed a correlation of 0.161 in CJD− samples, and 0.274 in CJD+ samples. Correlation between iron and Tf was 0.042 in CJD− samples as expected, and 0.319 in CJD+ samples (Figure 6A). Although the correlation between PrP and ferritin was also higher at 0.602 in CJD+ samples relative to 0.327 in CJD− samples, these observations could reflect the effect of astrogliosis with disease progression. Correlative values for TfR were not significantly different between the two groups. It is notable that the values for total iron are surprisingly similar in CJD+ samples, suggesting similar iron levels at end stage disease despite obvious differences in the background and disease course of each case (Figure 6A) (see Materials and Methods). Comparison of total brain iron in 55–66 year old CJD− patients carrying no definitive diagnosis with age-matched CJD+ cases shows a 1.4 fold increase of total iron in the latter (Figure 6B). Other CJD− samples, including cases of Alzheimer's disease, vascular disorders, dementia, and inflammation showed high levels of iron, each varying with the specific disease (Figure 6B). However, Tf and TfR levels did not show a corresponding decrease perhaps due to precipitation of iron in the extracellular space with little or no effect on cellular iron levels (Figures 3, 5C, and 6A). Linear regression analysis of the above data showed a positive correlation between PrP and Tf levels in CJD+ samples (R = 0.444), not in CJD− samples (R = 0.05) (Figure 6C). Immunostaining of brain sections revealed increased reactivity for Tf in cerebellar Purkinje cell neurons of CJD+ cases (Figure 7), indicating up-regulation of TfR expression in these cells. No visible deposits of iron were detected in any brain region of CJD+ cases, ruling out precipitation or co-aggregation of iron with PrP^Sc^ deposits (data not shown).

Increased levels of Tf, an iron uptake protein, in the presence of increased brain iron content observed above indicates imbalance of iron homeostasis in prion disease affected brains. A positive correlation between PrP and Tf levels further suggests that iron deficiency may occur as a result of PrP^Sc^ accumulation. Since a similar phenotype of iron deficiency is detected in human biopsy samples, alteration of brain iron homeostasis appears to occur before end-stage disease. It is notable that Tf levels are unlikely to be affected by secondary factors such as astrogliosis and neuronal loss, prominent features of end-stage prion disease [11],[12], and reflect neuronal iron status. In addition, our data indicate that Tf is a reliable marker of iron status in post-mortem tissue since it resists digestion by proteinase K and is likely to survive mild autolytic conditions.

### Imbalance of iron homeostasis increases with disease progression

**Figure 8:**
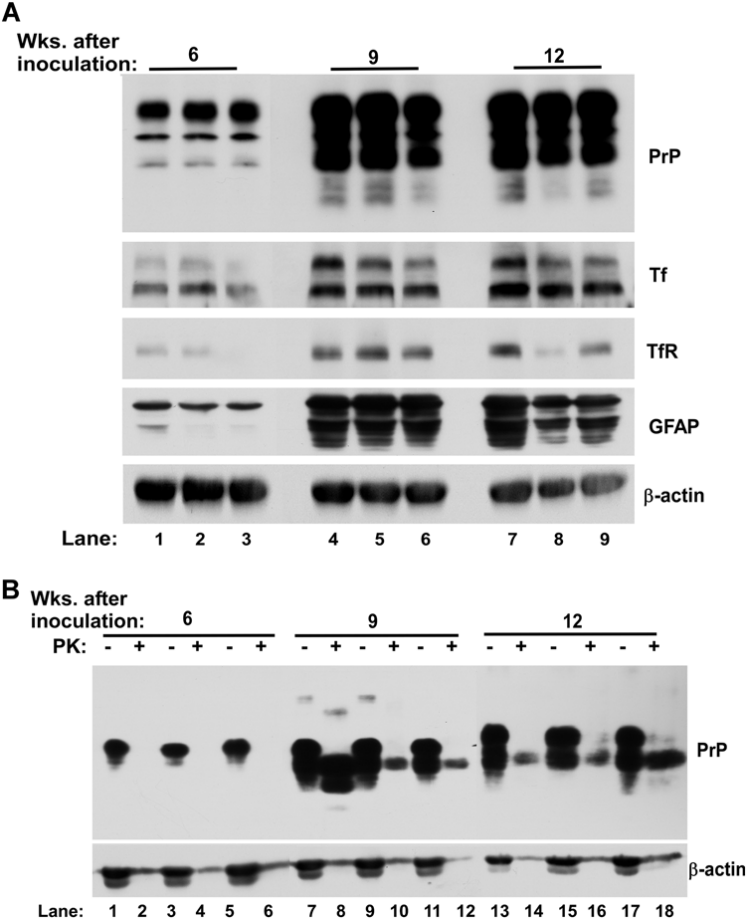
Scrapie-infected hamsters show increased iron deficiency with disease progression. (A) Immunoblot analysis of hamster brains harvested 6, 9, and 12 weeks after inoculation with 263 K strain of scrapie shows an abrupt increase in PrP, Tf, TfR, and GFAP expression after 9 weeks, and a further increase after 12 weeks (lanes 1–9). Reaction for β-actin shows protein loading for each sample. (B) The above homogenates were treated with PK and immunoblotted. Reaction for PrP shows the appearance of PK-resistant PrP^Sc^ 9 and 12 weeks post-inoculation (lanes 1–18). β-actin demonstrates protein loading, and is almost completely degraded by PK (lanes 1–18).

**Figure 9:**
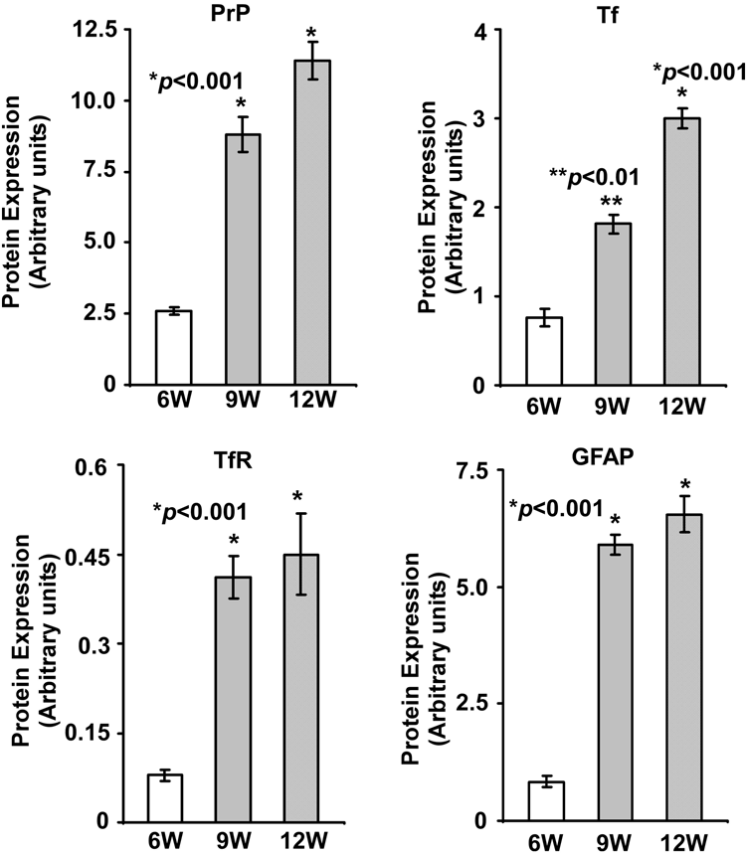
Densitometric analysis of protein levels in Figure 8. Quantification of protein levels in Figure 8A after normalization with actin shows a significant increase in PrP, TF, TfR, and GFAP levels at 9 and 12 weeks post-inoculation relative to the 6 weeks sample. Values are mean±SEM. *p<0.001, **p<0.01 relative to NH samples. n = 3.

**Figure 10:**
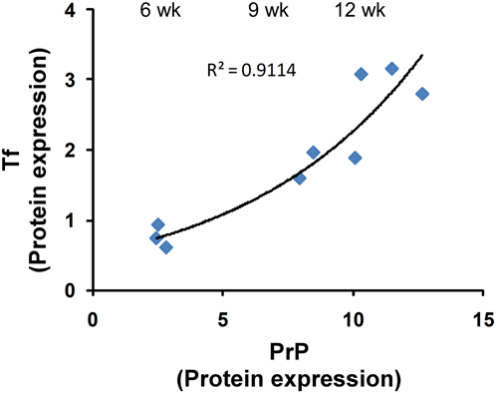
Correlation analysis of PrP and Tf. Association between PrP and Tf levels during disease progression was estimated by curve-fitting procedures. Exponential curve provided the best fit to the data indicated by R^2^ = 0.9114.

To evaluate the relationship between PrP^Sc^ accumulation and iron deficiency and to determine if imbalance of brain iron homeostasis appears earlier during the incubation period, hamster brain tissue harvested at 6, 9, and 12 weeks after inoculation with 263K strain of scrapie was analyzed. Immunoblot analysis showed a significant increase in PrP levels with disease progression as expected (Figure 8A, lanes 1–9). PK resistant PrP^Sc^ appeared 9 weeks after inoculation, and could be detected till 12 weeks, the end stage of disease (Figure 8B, lanes 1–18). Tf and TfR levels showed a significant increase at 9 weeks, and continued to increase till end stage of disease at 12 weeks post-inoculation (Figure 8A, lanes 4–9). Levels of GFAP, a marker of glial cells, showed a significant increase at 9 weeks and little change there-after (Figure 8A, lanes 4–9). Quantitative estimation of PrP, Tf, TfR, and GFAP levels in 9 and 12 week samples relative to 6 week post-inoculation samples revealed the following changes: PrP was increased by 3.4 and 4.4 fold, Tf was increased by 3.8 and 4.0 fold, TfR was increased by 5.2 and 5.7 fold, and GFAP was increased by 7.0 and 7.8 fold (Figure 9). Since Tf is a reliable indicator of brain iron status as demonstrated above, PrP and Tf levels at 6, 9, and 12 weeks post-infection were plotted to quantify the strength of association between the two proteins (Figure 10). Although limited data points suggest caution in interpretation, our results indicate highly significant and robust association indicating an exponential increase in Tf levels with increase in PrP expression (R^2^ = 0.91) (Figure 10). These results suggest that the rate of neuronal iron deficiency increases with PrP^Sc^ accumulation, with substantially higher increase between 9 to 12 weeks relative to that evidenced between 6 and 9 weeks. Since these hamsters are relatively young and lack other associated diseases that could contribute to these observations, our results suggest increasing iron deficiency with increase in PrP^Sc^ accumulation.

### Iron is sequestered in detergent-insoluble PrP^Sc^–ferritin complexes

**Figure 11:**
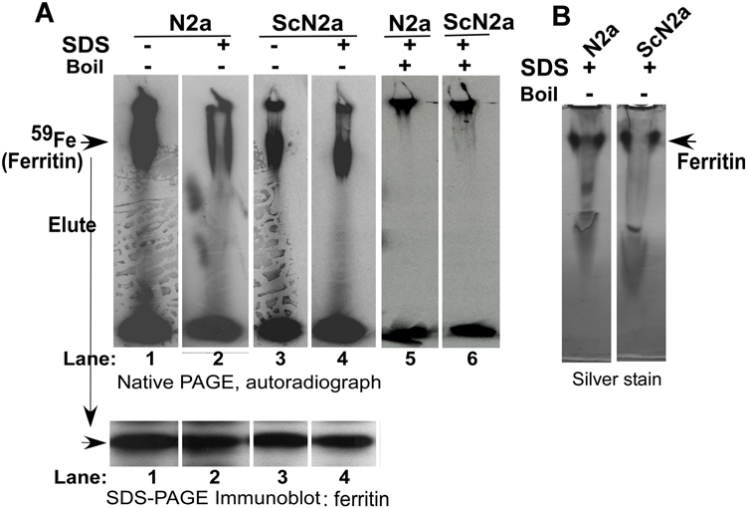
Iron is sequestered within ferritin in ScN2a cells and sCJD brains. (A) N2a and ScN2a cells cultured in the presence of 0.1 mM FAC were radiolabeled with ^59^FeCl_3_-citrate complex and lysed in native lysis buffer. Equal aliquots of the sample were either left aside, supplemented with 2% SDS, or supplemented with 2% SDS and boiled. All samples were resolved on a native gel followed by autoradiography. N2a lysates in native lysis buffer lacking SDS show significantly more incorporation of ^59^Fe into ferritin than ScN2a lysates (lanes 1 and 3). Following incubation with 2% SDS, a significant proportion of ^59^Fe dissociates from ferritin in N2a lysates, whereas the ScN2a sample shows little change (lanes 2 and 4). Boiling in SDS releases all ^59^Fe from ferritin in both N2a and ScN2a samples (lanes 5 and 6). Lower panel: Iron labeled band from N2a and ScN2a samples was eluted with boiling SDS and re-fractionated on SDS-PAGE, followed by immunoblotting with anti-ferritin antibody. The eluted proteins react strongly for ferritin, as described in a previous report [19]. (B) Silver staining of the same samples fractionated in (A) shows similar levels of ferritin following incubation with SDS, indicating only a change in iron content by this treatment.

It is plausible that a state of iron deficiency despite elevated iron in diseased brains arises from sequestration of iron in biologically unavailable PrP^Sc^-ferritin aggregates as suggested in a previous report [18]. To evaluate this possibility, N2a and prion infected ScN2a cells were exposed to 0.1 mM ferrous ammonium citrate (FAC) overnight to up-regulate ferritin expression. Treated cells were radiolabeled with ^59^FeCl_3_-citrate complex for 4 hours and chased in normal medium to accumulate ^59^Fe within ferritin. Cells were lysed in native lysis buffer and analyzed as such, or supplemented with 1% SDS for 10 minutes without boiling (Figure 11A, lanes 1–4) or with boiling (Figure 11A, lanes 5 and 6). Subsequently, the samples were separated by native gel electrophoresis followed by autoradiography. A prominent iron labeled band was detected in both N2a and ScN2a lysates (Figure 11A, lanes 1 and 3). (This band represents iron loaded ferritin as determined by re-fractionation of eluted proteins from the labeled band on SDS-PAGE followed by immunoblotting with anti-ferritin antibody (Figure 11A, lanes 1–4, lower panel)). Exposure to SDS dissociated a significant amount of iron from ferritin in N2a lysates (Figure 11A, lanes 1 and 2), but had little effect on the iron content of ferritin in ScN2a lysates (Figure 11A, lanes 3 and 4). Boiling in the presence of SDS released all iron from ferritin in both N2a and ScN2a lysates (Figure 11A, lanes 5 and 6). Silver staining of lanes 2 and 4 showed insignificant difference in ferritin protein levels in SDS treated N2a and ScN2a cells (Figure 11B), indicating that the difference arose from the iron content of ferritin. These results suggest that iron is sequestered in ‘SDS-insoluble’ ferritin aggregates in ScN2a cells, most likely in association with PrP^Sc^. Similar results were obtained with scrapie infected SMB cells, another prion infected cell line (data not shown) [23]. It is notable that incorporation of iron in ferritin from N2a cells is significantly more compared to ScN2a cells (Figure 11A, lanes 1 and 3), probably due to inaccessibility of iron to aggregated ferritin in the latter.

**Figure 12:**
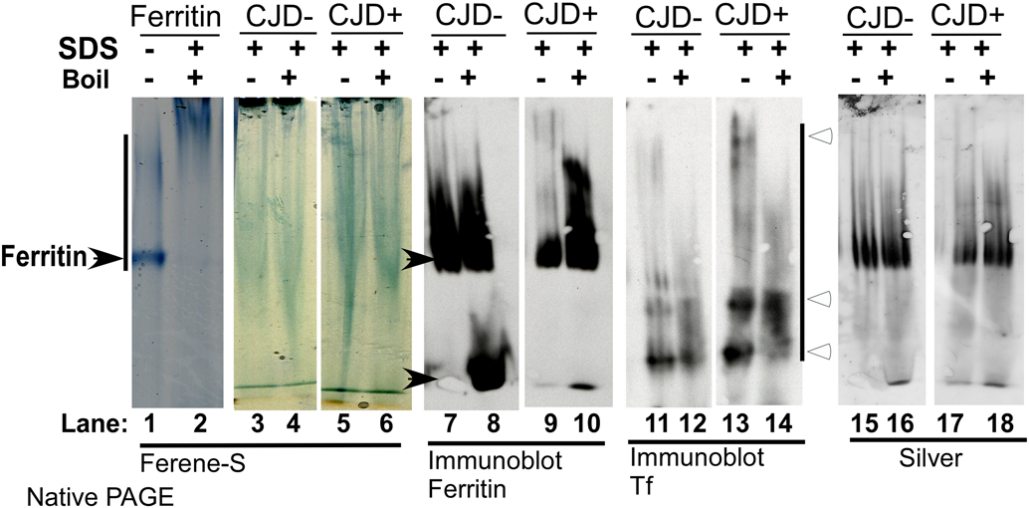
Ferritin from sCJD brain tissue is detergent-insoluble and retains iron. Commercially available purified ferritin and homogenates from CJD− and CJD+ brains were supplemented with 2% SDS and either left at room temperature, or boiled before resolving on a non-denaturing gel. The iron contained in purified ferritin reacts strongly with Ferene-S, but is lost after boiling (lanes 1 and 2). Relative to CDJ− samples, CJD+ samples show more reactivity with ferene-S even after boiling with SDS (lanes 3–6). Immunoblotting with ferritin shows a strong reaction in both CJD− and CJD+ samples. However, boiling in SDS denatures a significant amount of ferritin in the CJD− samples, while it has no effect on the CJD+ sample (lanes 7–10, black arrowheads). Reprobing for Tf shows relatively stronger reaction in the CJD+ sample, and partial denaturation by SDS in both samples (lanes 11–14, open arrowheads). Silver staining of the same membranes confirms equal loading of protein in all samples (lanes 15–18).

Since PrP^Sc^ from CJD+ brain homogenates is several-fold more resistant to PK digestion than PrP^Sc^ from ScN2a and SMB cells, a similar evaluation was carried out on CJD− and CJD+ brain homogenates. Thus, commercially available purified ferritin, CJD− and CJD+ brain homogenates were suspended in native lysis buffer or supplemented with 2% SDS and boiled for 10 minutes before separating on a non-denaturing gel. In-gel staining of separated proteins with the iron binding dye Ferene-S revealed a sharp blue band of iron in purified ferritin as expected, which was lost on boiling due to release of iron from denatured ferritin (Figure 12, lanes 1 and 2, respectively). A similar evaluation of CJD− and CJD+ samples showed more iron in CJD+ samples relative to CJD− controls under both conditions (Figure 12, lanes 3–6). A parallel set of samples was transferred to a PVDF membrane under non-denaturing conditions and probed for ferritin. Treatment of CJD− samples with boiling SDS denatured ferritin, some of which migrated with the dye front (Figure 12, lanes 7 and 8). A similar treatment of CJD+ samples had minimal effect on ferritin that migrated as oligomers without boiling, and as a relatively compact band after boiling (Figure 12, lanes 9 and 10, respectively). Re-probing for Tf revealed relatively higher levels in the CJD+ sample, though the bands did not react with Ferene-S, perhaps due to denaturation by SDS (Figure 12, lanes 11–14). Silver staining of the same membrane revealed equal loading of protein in all samples (Figure 12, lanes 15–18).

**Figure 13:**
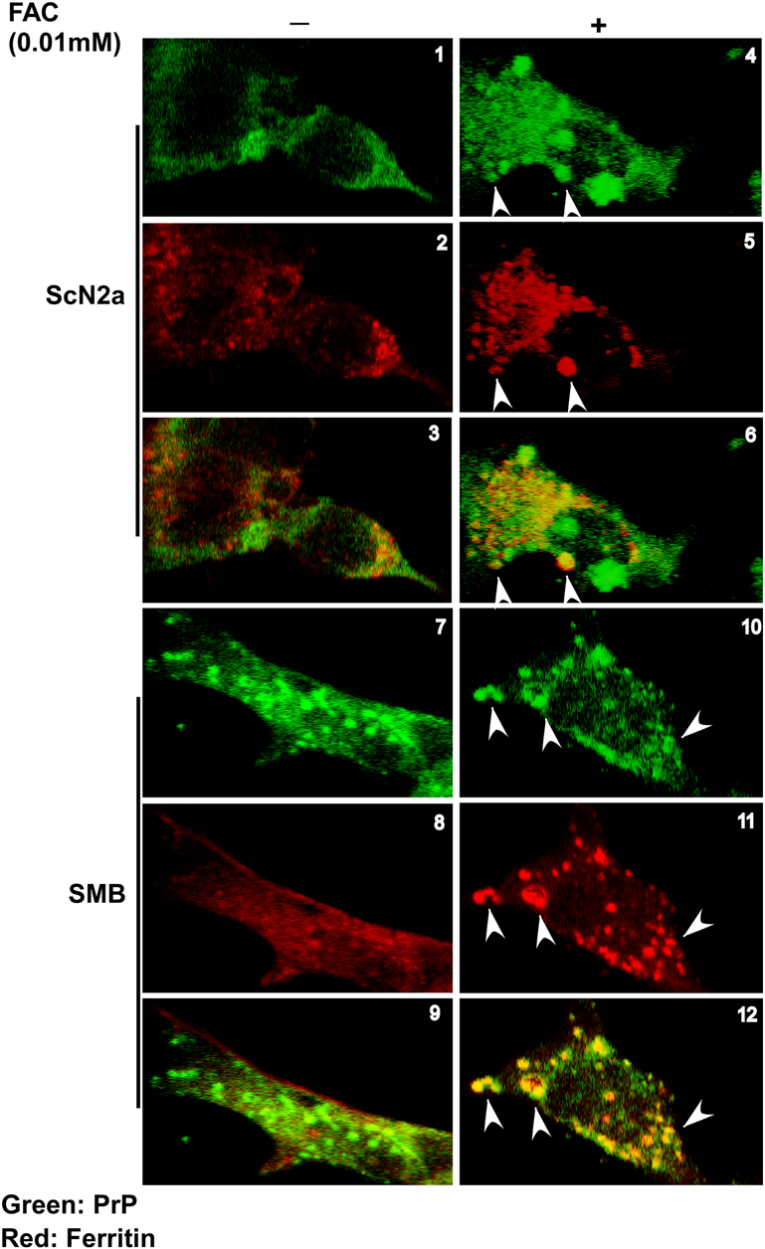
Scrapie-infected cells show co-localization of PrP and ferritin. ScN2a and SMB cells cultured in the absence or presence of 0.1 mM FAC were immunostained with 8H4-anti-mouse-FITC followed by anti-ferritin–anti-rabbit-TRITC. Untreated ScN2a cells show minimal PrP reactivity and reaction for ferritin in the cytosol (panels 1–3). Following exposure to FAC, both PrP and ferritin form intracellular aggregates that co-localize for the most part (panels 4–6, arrowheads). Untreated SMB cells show intracellular aggregates of PrP and reaction for ferritin in the cytosol (panels 7–9). However, exposure to FAC increases the reactivity for PrP and ferritin significantly, and, notably, PrP and ferritin form intracellular aggregates that co-localize in vesicular structures (panels 10–12, arrowheads).

To evaluate the cellular site where PrP^Sc^ and ferritin associate, ScN2a and SMB cells [22],[23] analyzed in Figure 11 above were immunostained for PrP and ferritin and examined (Figure 13). In contrast to control ScN2a cells that displayed minimal reaction for PrP and cytoplasmic staining for ferritin (Figure 13, panels 1–3), FAC exposed ScN2a cells revealed prominent intracellular reaction for PrP and ferritin, and significant co-localization of the two proteins (Figure 13, panels 4–6). Scrapie infected SMB cells showed significant intracellular reaction for PrP and minimal reaction for ferritin at steady state (Figure 13, panels 7–9). However, exposure to FAC revealed prominent intracellular vesicles that reacted for both PrP and ferritin (Figure 13, panels 10–12).

Together, the above results indicate that PrP^Sc^ and ferritin form detergent insoluble aggregates that sequester iron, creating a state of relative iron deficiency in prion infected cells.

## Discussion

In this report we demonstrate that prion infection induces a state of iron imbalance in diseased human, hamster, and mouse brains as revealed by a phenotype of iron deficiency in the presence of increased total iron. The phenotype of iron deficiency appears early in the disease process, probably with the initiation of PrP^Sc^ formation, and is not an outcome of end-stage disease. An important underlying cause of iron imbalance is sequestration of cellular iron in detergent insoluble PrP^Sc^-ferritin complexes, thus rendering it bio-unavailable and creating a phenotype of apparent iron deficiency. Consequent up-regulation of iron uptake proteins Tf and TfR result in increased cellular iron uptake, creating a state of cellular iron imbalance. Since iron is potentially toxic due to its redox-active nature, these observations have significant implications for prion disease associated neurotoxicity.

In evaluating iron homeostasis in healthy and diseased brains, we have focused primarily on total brain iron and levels of Tf and TfR. Although these parameters do not represent changes in iron levels in individual cell types that comprise the complex milieu of the brain, these are reliable markers of neuronal iron levels, the principal cell type affected in prion disorders [14]. The mechanisms underlying brain iron homeostasis are complex, and details of iron import and export from various cells within the brain are still emerging. Normally, iron enters the brain through the Tf/TfR pathway across capillary endothelial cells, and is delivered to the brain interstitium using mechanisms that are still debated. Once in the brain, iron is bound by citrate, ascorbate, and Tf present in the interstitial fluid. Citrate and ascorbate are mainly released by astrocytes, and brain Tf is derived from cells of the choroid plexus and oligodendrocytes and is saturated with iron under normal conditions. Neurons take up most of their iron from Tf through TfR mediated uptake. Under conditions of iron deficiency, neurons up-regulate TfR levels to internalize increased amounts of iron saturated Tf to make up for the deficiency. The opposite scenario holds true when iron is in excess. In contrast to other cell types such as astrocytes, oligodendrocytes, and microglia, neurons in most brain regions lack the iron storage protein ferritin, necessitating efflux of excess iron through the iron export protein ferroportin. Astrocytes, microglia, and oligodendrocytes lack TfR, and take up most of their iron through other mechanisms [2], [14]–[16]. Thus, TfR levels in the brain reflect the iron status of neurons, the most vulnerable cell population in prion disorders. However, experimental manipulation of brain tissue often results in the degradation of TfR, providing inconsistent results from post-mortem brains and animal brains unless the tissue is handled with extreme care. Tf, on the other hand, is as resilient to degradation as PrP^Sc^, and reflects TfR levels as demonstrated by our immunohistochemistry and immunoblot data from animal and human brains. It is for these reasons that we have focused on Tf and iron levels, and where possible, TfR levels to evaluate brain iron status in prion disease affected brains.

Using the above criteria, our data demonstrate the presence of iron imbalance in prion disease affected brains from three different species, human, hamster, and mice. Although alteration of brain iron has been described in other neurodegenerative diseases such as AD and PD, it is notable that the changes in iron homeostasis in prion disease affected brains are distinct [1]–[4]. A prominent difference is the apparent phenotype of iron deficiency in prion disease affected brains despite increased brain iron content. A direct correlation between PrP and Tf levels in both sCJD and infected hamster samples suggests that iron deficiency arises as a direct consequence of PrP^Sc^ accumulation, perhaps by forming PrP^Sc^-ferritin complexes as noted in scrapie infected cells and reported earlier [18],[25]. An increase in Tf and TfR levels is also noted in human biopsy samples, indicating that brain iron levels decrease much before the onset of end-stage pathology. The presentation of AD and PD brains, however, is strikingly different. In the pool of CJD− cases examined in this report, several samples contained high levels of iron, including cases of AD, PD, dementia of unknown origin, and vascular disorders. As opposed to prion disease affected brains, these cases demonstrated a consistent down-regulation of Tf and TfR as expected, suggesting that the increased iron content was probably derived from insoluble deposits in the extra-cellular space. Brains from sCJD cases, on the other hand, show a modest and a surprisingly similar increase in iron content across twenty samples and a significant increase in Tf levels, suggesting a common pathway of iron accumulation and sequestration by scrapie infection.

The data on scrapie infected hamsters support the above conclusions. In these animals, iron deficiency appears almost at the same time as PK-resistant PrP^Sc^, and worsens with disease progression as indicated by increasing levels of Tf and TfR till end stage disease. Since this analysis was performed on experimental brains that were snap frozen after harvesting, the increase in TfR levels with disease progression provides a direct measure of neuronal iron deficiency, further supported by an increase in Tf that is likely to reflect TfR levels as observed in the Purkinje cell neurons of CJD+ samples. Since most neurons lack ferritin iron stores [14], such a situation is likely to result in compromised neuronal health and toxicity. It is unlikely that advanced age contributes to the iron imbalance observed in prion disease affected brains since the hamster brains used for this evaluation range in age from ten to sixteen weeks, a relatively young age considering the life-span of hamsters. It is interesting to note that the extent of iron accumulation varies with the strain of prions used for infecting the experimental animal. Hamsters infected with TME accumulate much higher levels of total and redox-active iron than those infected with the 263K strain of scrapie (unpublished observations). However, the increase in Tf levels is similar in both cases, suggesting that iron in some cases precipitates in the extra-cellular space and does not alter the intracellular iron status.

The most likely cause of iron deficiency in prion disease affected brains is the sequestration of iron in PrP^Sc^-ferritin aggregates as noted in scrapie infected cells and sCJD brain tissue. In a previous report we demonstrated that PrP^Sc^ from sCJD and scrapie infected mouse tissue forms a PK-resistant, detergent insoluble complex with ferritin [18],[25]. Here we show that ferritin from sCJD affected brains sequesters iron, and is not denatured by boiling in 2% SDS for ten minutes. Ferritin from normal human brains is denatured by this treatment and releases bound iron. A similar observation is noted in scrapie infected cell lines, though cellular ferritin is less resistant to SDS. These observations suggest that ferritin co-aggregates with PrP^Sc^ to form a detergent insoluble complex and sequesters the associated iron, creating a state of iron deficiency. Such an occurrence would explain the development of iron deficiency with the appearance of PrP^Sc^, increased levels of total iron in diseased brains, a direct correlation between PrP and Tf levels, and an overall state of iron imbalance in diseased brains. Although ferritin is mainly cytosolic, it is known to undergo degradation within the lysosomes, the cellular compartment where PrP^Sc^ and ferritin are observed in scrapie infected cells, and are hence likely to form the complex [18].

To summarize, this report demonstrates that brain iron imbalance is a common feature of prion disease affected brains, and is likely to contribute to prion disease associated neurotoxicity due to the redox-active nature of iron. The underlying cause of iron imbalance is the formation of PrP^Sc^-ferritin complexes, a pathogenic process that may lend itself to therapeutic manipulation, thus providing a means to reduce disease associated neurotoxicity.

## Materials and Methods

### Cell lines, chemicals, and antibodies

Mouse neuroblastoma cells (N2a) and ScN2a cells were obtained from Dr. Byron Caughey (Rocky Mountain laboratories), and scrapie infected SMB cells (mouse brain cells of mesenchymal origin were obtained from the TSE Resource Center, Institute of Animal Health (Edinburgh, Scotland). PrP-specific monoclonal antibodies 3F4 and 8H4 were obtained from Signet (Dedham, MA, USA) and Drs. Man-Sun Sy and Pierluigi Gambetti (National Prion Surveillance Center, Case Western Reserve University). Anti-ferritin antibody was from Sigma-Aldrich (St. Louis, MO), anti-Tf from GeneTex (San Antonio, TX) and anti-TfR antibody from Zymed Laboratories Inc (Carlsbad, CA). Horseradish peroxidase (HRP)-labeled secondary antibodies were from GE Healthcare (Little Chalfont, Buckinghamshire, United Kingdom). All other chemicals were purchased from Sigma.

### Brain tissue samples

Frozen human brain tissue from the frontal cortex of CJD+ and matched controls (CJD−) was obtained from the National Prion Surveillance Center, Case Western Reserve University. Additional samples of control tissue (CJD−) were obtained from the Harvard Tissue Bank. Collectively, the samples ranged in age from 37–80 years, and the disease duration ranged from 1–24 months in CJD−, and 2 to 4 months in CJD+ cases. CJD− cases carried the diagnosis of extensive infarcts, possible granulomatous angiitis, cerebral vascular disease, and necrotizing lymphoplasmacytic meningoencephalitis (1 case each), Alzheimer's disease (5 cases), and no definitive diagnosis (11 cases). CJD+ cases were classified as MM1 (11 cases), VV1 (2 cases), VV2 (4 cases), and MV2 (3 cases). Male and female representation was almost equal among the CJD− and CJD+ groups. Given the heterogeneity in the two groups, samples were matched only for age when loading on SDS-PAGE. For SDS-PAGE, 10% homogenate was prepared in lysis buffer (10 mM Tris, pH 7.5, containing 150 mM NaCl, 0.5% sodium deoxycholate, 0.5% nonident P-40 containing PMSF and Protease inhibitor cocktail). For determination of iron, the sample was prepared in PBS and analyzed using the Teco diagnostic kit as described below. Brain biopsy samples were obtained from the Prion Disease Surveillance Center at Case Western Reserve University, and represent individuals ranging in from 46 and 80 years. CJD− cases presented with positive neurological symptoms for 1–12 months, and CJD+ cases for 2–4 months. Due to the limited amount of tissue available from biopsies, PVDF membranes used for diagnostic purposes were re-probed to evaluate the expression of major iron regulatory proteins ferritin, Tf and TfR.

Control and TSE infected end stage hamster brain tissue was obtained from Jason Bartz (Department of Medical Microbiology and Immunology, Creighton University, Omaha, Nebraska) [21] was analyzed as above for protein analysis, and homogenized in PBS for estimating total, Fe(II), and (Fe(III) iron [26]. To evaluate the point of initiation of iron dyshomeostasis during disease progression, hamster brain tissue collected at 6, 9, and 12 weeks after inoculation with 263K strain of scrapie was obtained from Qingzhong Kong (Case Western Reserve University). Half brain from each sample was homogenized in lysis buffer analyzed for PK resistant PrPSc as well as expression of other proteins. Control and scrapie infected end stage mouse brain tissue (strains 137A and 22L) was generated in our facility and obtained from Jason Bartz and analyzed as above.

### Proteinase-K digestion, SDS-PAGE, and Western blotting

Normal (NH) and diseased brains homogenized in lysis buffer (10 mM Tris, pH 7.5, containing 150 mM NaCl, 0.5% sodium deoxycholate, 0.5% nonident P-40) were incubated with 50 and 100 µg/ml of PK at 37°C for h. Digestion was stopped by addition of 1 mM PMSF and protease inhibitor cocktail. Digested and undigested samples were processed for SDS-PAGE and Western blotting as described previously [27],[28]. Membranes containing transferred proteins were probed with anti-PrP antibodies 3F4 (1∶5000) or 8H4 (1∶3000), anti-ferritin (1∶1000), anti-Tf (1∶6000), anti-TfR (1∶3000), anti-GFAP (1∶2000), and anti-β-actin (1∶7500) followed by appropriate secondary antibody conjugated with horseradish peroxidase (1∶6000). Immunoreactive bands were visualized by ECL detection system (Amersham Biosciences Inc.).

### Determination of iron in brain homogenates

A colorimetric method (Teco Diagnostics, Anaheim, CA) was used to estimate total iron in brain homogenates according to the manufacturer's instructions. In short, 20 µl of 10% homogenate in PBS was dissolved in 1 ml of acetate buffer containing 220 mM Hydroxylamine hydrochloride, pH 4.5 and 50 µl of 16.6 mM Ferrozine was added to develop the color. The change in color was recorded at 560 nm after 10 minutes of incubation at 37°C using 500 mg/dl FeCl_2_ solution as standard. Negative controls were recorded without the addition of ferrozine and background was subtracted from test samples.

### Radiolabeling with ^59^FeCl_3_ and native gradient gel electrophoresis

Mouse neuroblastoma cells (N2a) and scrapie infected ScN2a and SMB cells were cultured in the absence or presence of 0.1 mM ferrous ammonium citrate and labeled with ^59^FeCl_3_-citrate complex (1 mM sodium citrate and 2 µCi of ^59^FeCl_3_ in serum free Opti-MEM) overnight at 37°C in 5% CO_2_ in a humidified atmosphere. Washed cells were lysed in native lysis buffer (0.14 M NaCl, 0.1 M HEPES, pH 7.4, 1.5% Triton X-100 and 1 mM PMSF), and half of each sample was supplemented with 10 µl of 10% SDS for 10 minutes at room temperature. Both parts were mixed with glycerol, traces of bromophenol blue, and resolved on 3–9% native gradient gel as described by Petrak and Vyoral [29],[30] with modifications [19]. Gels were vacuum dried and exposed to X-ray film to visualize labeled bands. A duplicate set of samples was analyzed on native gel, and the radioactive band was eluted with boiling SDS and re-fractionated on a SDS-PAGE gel followed by immunoblotting with anti-ferritin antibody.

### Detection of iron on native gel with Ferene S

Brain homogenates prepared in 5 mM HEPES pH 7.4, 0.25 M sucrose and protease inhibitors were resolved on native gradient gels (3–9%) without Triton X-100 and stained for iron with freshly prepared Ferene-S (0.75 mM 3-[2-pyridyl]-5, 6-bis (2-[-furyl sulfonic acid]-2, 4-triazine, 2% (v/v) acetic acid, 0.1% thioglycolic acid) [24] for 30 min at 37°C. A dark blue stain indicates the presence of iron or iron-bound protein. The identity of iron stained bands was confirmed by immunoblotting with specific antibodies.

### Detection of redox-iron

Prussian blue reaction was used to detect redox-active ferrous and ferric iron in brain homogenates as described in [26].

### Immunostaining of brain sections

Paraffin embedded cerebellar sections of CJD+ cases and age matched controls were deparafinized with methanol followed by exposure to 8% H_2_O_2_ and water. Cleared sections were incubated with 10% NGS followed by anti-Tf antibody (1∶20 in 1% NGS) overnight at 4°C. After washing with TBS the sections were incubated with peroxidase-conjugated secondary antibody for 60 min and developed with DAB.

### Immunostaining and fluorescence microscopy

N2a and SMB cells exposed to FAC were processed for immunostaining as described in a previous report [18].

### Analysis of iron–PrP^Sc^–ferritin aggregates from sCJD brain homogenates

Human brain tissue from CJD+ and matched controls (CJD−) homogenized in PBS was used for the analyses. Brain homogenate was mixed with native lysis buffer (0.14 M NaCl, 0.1 M Hepes, 1.5% Triton X-100, 1 mM PMSF, PH 7.4), or supplemented with 2% SDS. Samples were either incubated at 37°C or boiled for 10 min and resolved by 3–9% native gradient gel. Iron levels were detected by Ferene S staining as described above. To confirm the identity of ferritin and Tf, proteins were immunoblotted under native conditions as described previously [19] and membranes were probed for ferritin and Tf. Equal loading was confirmed by silver staining of membranes.

### Statistical analysis

Data are presented as the mean SEM values. Statistical evaluation of the data was performed by using Students t-test (unpaired). Regression analysis was performed by standard statistical methods and the Stata (2005) statistical package.
